# An integrated mental health and vocational intervention: A longitudinal study on mental health changes among young adults

**DOI:** 10.1002/nop2.560

**Published:** 2020-07-09

**Authors:** Ulrika Liljeholm, Elisabeth Argentzell, Ulrika Bejerholm

**Affiliations:** ^1^ Department of Health Sciences/Mental Health, Activity and Participation Lund University Lund Sweden; ^2^ Centre for Evidence‐based Psychosocial Interventions Lund University Lund Sweden

**Keywords:** bipolar disorder, community integration, major depressive disorder, psychiatric rehabilitation, recovery, vocational rehabilitation

## Abstract

**Aim:**

This study aimed to investigate changes in mental health among young adults participating in an integrated mental health and vocational support intervention according to the Södertälje Supported Employment and Education model.

**Design:**

A prospective longitudinal pre–post intervention study of 12 months.

**Methods:**

Instruments on depressive symptoms, quality of life, empowerment, engagement in activities and sociodemographic characteristics were administered to 42 young adults aged 19–28 years with mood disorders. Wilcoxon signed rank tests were used to assess changes in mental health.

**Results:**

Statistically significant positive changes between baseline and 12 months were noted for quality of life and engagement in activities. Difference in empowerment scores neared significance and a statistical trend towards lower depression scores was seen, corresponding to moderate depression at baseline and less severe depression at 12 months.

**Conclusions:**

Integrated mental health and vocational services may support young adults' mental health and is suggested to be linked to their personal recovery and clinical recovery.

## 1 INTRODUCTION

The increase in mental health problems among young adults has become a serious public health challenge (National Board of Health & Welfare, 2013). During 2000–2010, the number of young adults who had contact with mental health services in Sweden increased by about one third. In 2016, alarming statistics were seen. Fifteen per cent of young women and 10% of young men aged 18–24 years needed some form of psychiatric treatment (National Board of Health & Welfare, 2017; Social Insurance report, 2014). Depressive disorders, different anxiety disorders and addiction are the diagnoses that have increased most rapidly (National Board of Health & Welfare, 2017). Mental health problems at a young age are associated with social, occupational or other important areas of functioning (American Psychiatric Association, 2013; Swan & Kendall, 2016). This situation prevents the development of important changes associated with adulthood (Babajide, Ortin, & Wei, 2019; Copeland, Angold, Shanahan, & Costello, 2014; Scott, Scott, & Hermens, 2014; Swan & Kendall, 2016) and thus influences educational achievement, transition to work and have serious consequences later in life. These include a lower level of education and problems in attaining a working life (National Board of Health & Welfare, 2013; Swan & Kendall, 2016). For young adults, mental health problems may become an obstacle for career development, transition to adulthood and the ability to provide for themselves through employment (National Board of Health & Welfare, 2013). However, work is a common goal for persons with mental health problems (Bedell, Draving, Parrish, Gervey, & Guastadisegni, 1998; McQuilken et al., 2003). For those who dropped out of school, more than half wanted to return to studies to make changes and improve their work status (Corrigan, Barr, Driscoll, & Boyle, 2008; Knis‐Matthews, Bokara, DeMeo, Lepore, & Mavus, 2007). Furthermore, work has shown to have an essential importance for mental health changes (Bejerholm & Areberg, 2014; Boardman, Grove, Perkins, & Shepherd, 2003; Dunn, Wewiorski, & Rogers, 2008; Provencher, Gregg, Mead, & Mueser, 2002). Also, work identity development is essential for the personal recovery process of young adults with mental health problems (Liljeholm & Bejerholm, 2019). Therefore, the availability of early efforts that target counteraction of long‐term mental health problems and help with employment prospects need to be improved. In previous research, the importance of providing appropriate mental health services that considers mental health changes in young adults in a timely manner is clearly emphasized (Babajide et al., 2019; Copeland et al., 2014; Scott et al., 2014; Swan & Kendall, 2016).

The concept of mental health includes mental, psychological and social well‐being components that affect how a person thinks, feels, acts and makes life choices (World Health Organization, 2003). Mental health contains both the person's own experience of well‐being and the relationship between the person and his or her social context (Bremberg & Dalman, 2015). Consequently, a person's opportunities for social life and community integration activities influence his or her mental health. This is a reflection of the concept that mental health focuses on clinical (symptoms) and personal recovery (meaningful life roles) and is vital to a person‐centred care approach (Anthony, 1993; Bejerholm & Björkman, 2011; Bird et al., 2014). Having depressive symptoms may worsen mental health and impact mood, energy and activity. These effect a person's ability to achieve meaningful life roles related to work, school or coping with daily life (World Health Organization, 2017). The quality‐of‐life concept in mental health is linked to general satisfaction through various life domains, such as work status, private finances, relationships with family and friends, housing and leisure activities (Bejerholm, Hansson, & Eklund, 2006; Priebe, Huxley, Knight, & Evans, 1999). Perceived quality of life in relation to a person's mental health is therefore vital to study. Furthermore, perceived control over several life domains and being empowered are shown to be crucial for a person's experience of quality of life (Corrigan, 2006; Lloyd, King, & Moore, 2010; Warner, 2009). To be empowered includes a psychological dimension that refers to an internal strength of self‐efficacy and self‐esteem and having the opportunity and ability to make one's own important life decisions (Corrigan, 2004). In a previous grounded theory study on work identity development, the transition to work and career development was shown to be critical for young adults' self‐efficacy and future outlook through influencing more positive thought patterns (Liljeholm & Bejerholm, 2019). If increased empowerment can counteract depressive symptoms, then to capture mental health changes, studying perceived quality of life and the internal strength of empowerment is necessary. To study mental health more fully, a focus on the level of engagement in everyday life activities is needed.

International policies emphasize that mental health services should not exclusively focus on clinical recovery with the aim of improving function and lessening or managing symptoms and difficulties (Bejerholm & Roe, 2018; Bellack & Drapalski, 2012; Swedish Governments Official Investigations, 2006). In such circumstances, there is a risk that service users remain in a patient role and do not develop meaningful life roles such as the worker role. This study focused on an intervention with a personal recovery perspective that includes the possibility of developing meaningful life roles despite having mental health symptoms and problems (Anthony, 1993). From this perspective, mental health services are challenged to adopt a person‐centred care to increase the chance for both clinical and personal recovery to improve mental health and the situation for this valued group of young adults (Anthony, 1993; Bejerholm & Björkman, 2011; Bergmark, Bejerholm, Svensson, & Markström, 2018; Bird et al., 2014). Person‐centred here refers to working together with service users in equal partnership to reach solutions according to their interests, needs, values, social situation and life circumstances (Leamy, Bird, Boutillier, Williams, & Slade, 2011). It means that services need to be accessible, flexible and integrated around user goals (Brown, 2013; McCance, McCormack, & Dewing, 2011). To meet this recovery ideal, the mental health and vocational service was merged into one intervention and evaluated within the group of unemployed young adults with mental health problems.

According to official Swedish Governments Investigations (2018), two of the greatest risk factors for unequal conditions in community are mental health problems and unemployment. Previous research has shown that integrated mental health and vocational services, that is supported employment, that aimed to support competitive employment among adults (mean age 40 years) with mental health problems resulted in gains in acquiring and keeping employment (Bejerholm, Larsson, & Johanson, 2017) and a greater sense of empowerment (Porter & Bejerholm, 2018). The service users also experienced fewer depressive symptoms and higher levels of engagement in everyday life activities compared with those in traditional services that focused separately on clinical and personal recovery (Areberg & Bejerholm, 2013; Areberg, Björkman, & Bejerholm, 2013; Johanson, Bejerholm, & Markström, 2017; Porter, Lexén, Johansson, & Bejerholm, 2018). Previous research shows that vocational efforts that lead to sustainable career development for persons with mental health problems are essential for personal recovery (Bejerholm & Areberg, 2014; Boardman et al., 2003; Dunn et al., 2008; Provencher et al., 2002). Research is still scarce about how integrated mental health and vocational services may influence young adult (aged 18–29 years) mental health. Although international research forward the importance of work for young adults' self‐development and social engagement (Lloyd & Waghorn, 2007; Torres Stone, Sabella, Lidz, McKay, & Smith, 2018; Young, Ng, Cheng, & Leung, 2018), which underlines the necessity to integrate vocational and mental health services into an overall intervention.

A previous related grounded theory study reflected the impact the person‐centred and joint mental health and vocational intervention made. It showed that opportunities to enter into social life and the context of a workplace function as a constructive, productive, activity arena where a work identity process can be developed and self‐efficacy is enhanced (Liljeholm & Bejerholm, 2019). Having person‐centred support in reaching meaningful personal recovery goals such as work and getting involved in work may be critical for positive changes in mental health. This is an explorative and prospective longitudinal study that aimed to investigate clinical and personal recovery‐related outcomes in mental health among young adults who receive a new intervention of integrated mental health and vocational services for 12 months.

## 2 MATERIALS AND METHODS

### 2.1 Design and context

A prospective longitudinal and pre–post intervention design collected baseline, six‐ and 12‐month follow‐up data. The study sample included 42 young adults who were service users in mental health services and wished to enter working life. The research context was a mental healthcare unit with a focus on integrated services for young adults in the city of Södertälje (*N = *32, ages 18–23). The project and present study were approved by the Ethical Review Board (Reg. No. 2014‐277). To increase the sample size and age range of 18–29 years, which is in harmony with the age range of defining young adults with a disability in the Swedish welfare system (Public Employment Service, 2014), a subsample (*N = *10, ages 20–28 years) from a research project focusing on ages 18–65 in Skåne County Council was included (Reg. no. 2011‐544). Both contexts had the same principal investigator (last author) and used the same eligibility criteria, data collection methods, education and clinical supervision. While the person‐centred and integrated model was novel in the Skåne county council, the city of Södertälje in Sweden has had a long record of providing flexible and integrated mental health (county council) and social care services (municipality), including vocational services (Klinga, Hasson, Sachs, & Hansson, 2018). The innovative way of integrating welfare services in Södertälje has further stated an example for how to implement supported employment and supported education in a Swedish context. It was therefore decided by the research team to name the current intervention the Södertälje Supported Employment and Education model (SSEE) hereafter.

### 2.2 Participants

Eligibility criteria included ages 18–29 years, able to communicate in Swedish, had expressed an interest in employment, had received mental health services and had major depressive or bipolar disorder. Diagnoses were set by the team psychiatrist according to the Diagnostic and Statistical Manual of Mental disorders 5th edition (American Psychiatric Association, 2013). All participants (*N = *42) attended a research informational meeting where they were given verbal and written information about voluntary participation, use of data in relation to the research aims, confidentiality protections and ability to withdraw from the study at any time, according to the principle of autonomy (Beauchamp & Childress, 2013). Written informed consent was obtained from each participant.

### 2.3 Data collection

Data collection took place at the mental health service setting and lasted for 1–2 hr at baseline and one hour at follow‐ups. Two trained research assistants, with no earlier relationship to the participants, conducted and guided the participants during data collection. The research assistants had extensive experience of working with the target group and the instrument used.

Several instruments were used at three measurement points (baseline, six and 12 months) to measure changes in mental health. One questionnaire targeted sociodemographic characteristics such as age, gender, employment status, educational level, housing situation and clinical data on diagnosis and care use. Other instruments presented below were included to address clinical and personal recovery outcomes.

Mental health changes in terms of clinical recovery and symptom outcomes of were assessed using the self‐rated Montgomery–Åsberg Depression Rating Scale (MADRS‐S). This instrument consists of nine items: mood, feelings of unease, sleep, appetite, ability to concentrate, initiative, motivational involvement, pessimism and zest for life. The sum score ranges from 0–54 and is categorized as no or hardly any depression (0–12), less severe depression (13–19), moderate depression (20–34) and severe depression (≥35) (Montgomery & Asberg, 1979). MADRS‐S has satisfactory construct validity, internal consistency, reliability and sensitivity to change (Fantino & Moore, 2009). It is used as a routine outcome measurement in mental health services. Cronbach's alpha in the current sample was *α* = 0.768.

Mental health changes in terms of personal recovery‐related outcomes were assessed by means of various instruments. To begin with, quality of life was assessed with Manchester Short Assessment of Quality of Life (MANSA). MANSA is a condensed instrument for assessing quality of life through perceived satisfaction within 12 life domains. The instrument is suitable for use in cross‐sectional studies and longitudinal studies (Björkman & Svensson, 2005) and is used as a routine outcome measurement in mental health services. The instrument consists of 16 questions that cover different life domains such as work, finances, social networking, leisure activities, housing, personal safety, family relationships, sex life and physical and mental health. Twelve questions are strictly subjective and have satisfaction values between 1–7, where 1 means “could not be worse” and 7 is “could not be better.” The sum range may score from 16–84. A mean of 4.75 is interpreted as good quality of life (Priebe et al., 1999). MANSA is generic and has sound psychometric properties (Priebe et al., 2012). Cronbach's alpha in the current sample was *α* = 0.84.

Empowerment was measured using the Empowerment Scale (ES). The instrument was developed by Rogers, Chamberlin, Ellison, and Crean (1997) and includes 28 statements that are organized into five subscales: self‐esteem/self‐efficacy, power/powerlessness, community activism and autonomy, optimism and control over the future and righteous anger. Individuals grade their estimation from 1 (strongly agree)–4 (strongly disagree). The sum score may range from 28–112, with a higher score indicating greater perceived empowerment (Rogers et al., 1997). The ES instrument has sound psychometric properties, and the Swedish version has good internal consistency (Hansson & Björkman, 2005). Cronbach's alpha in the current sample was *α* = 0.88.

Time‐use diaries were used to measure the daily rhythm of activity and rest, meaningful activities, social interplay, the ability to reflect and make sense of experiences, time spent in social and geographical environments, taking initiative and having daily routines (Bejerholm & Eklund, 2007). Engagement in everyday life was measured by means of Profiles of Occupational Engagement in people with Severe mental illness (POES). POES consists of two parts. The first is a 24‐hr time‐use diary with one‐hour intervals and three columns. The participants fill in their activities, social and geographical environment and reflections and reactions connected to the activity experience. In the second part, participants assess their completed time‐use diary according to the nine items on a four‐point scale. This later part addresses the extent to which the informant has a balanced daily rhythm of activity and rest, time spent in a variety of geographical and social environments, variety and range of activities, management of social interplay, reflection on occupational experience, experience of meaningful occupations, presence of routines and initiation of own activities (Bejerholm et al., 2006). The sum score ranges from 9–36, with a higher score indicating a higher level of engagement. POES is not diagnosis‐specific but rather is generic and has good psychometric properties (Bejerholm & Eklund, 2006; Bejerholm & Lundgren‐Nilsson, 2015). Cronbach's alpha in the current sample was *α* = 0.81.

### 2.4 Intervention

All participants received the SSEE model for 12 months. Since supported employment is recognized as a recovery‐oriented intervention that focuses on both clinical and personal recovery (Bejerholm & Roe, 2018), the mental health and vocational services were integrated in a similar manner. This forms a critical part of the supported employment principles: (a) employment is the goal; (b) vocational services is integrated with mental health services; (c) eligibility is based on service user's desire to work; (d) personalized benefit counselling; (e) rapid job search; (f) systematic development of relationship with employers; (g) continuous support; and (h) service user's interests, preferences and needs are honoured (Drake, Bond, & Becker, 2013). Either an employment specialist or a case manager took on the key professional role in the outpatient team to integrate individuals work goals according to their preferences, interests and support needs. To support improvement of mental health changes in young adults, it seems critical to provide a person‐centred mental health service where work‐related aims are adopted and forwarded. Such interventions have previously been found to support the experience of self‐esteem, empowerment and engagement in everyday activities (Liljeholm & Bejerholm, 2019; Young et al., 2018) and community life in general (Bejerholm & Roe, 2018; Bellack & Drapalski, 2012).

The intervention delivery was approximately one hour per week. This intervention entailed person‐centred support and planning that paid attention to the individual's personal resources and efforts to live a meaningful life (Bejerholm & Roe, 2018; Bellack & Drapalski, 2012). The intervention began with talking through the individual's needs, resources and preferences in relation to starting to work and integrating joint mental health and vocational support and planning job development activities. Further, the interventions focused on enabling motivation for work and different lifestyle strategies related to this, as well as developing a career profile and plan and helping with job seeking. When the individual gained employment, the intervention continued, with individual support at work. A support network, that is family, friends, Social Insurance Agency, Public Employment Service and employers, was also mobilized as needed to address a sustainable work situation.

### 2.5 Statistical methods

Descriptive statistics were used for sociodemographic and clinical characteristics for the participants. Non‐parametric statistics were used for calculations of the data. Non‐parametric statistics were viewed as the most appropriate since the data were of an ordinal and categorical nature and non‐normally distributed. Furthermore, since the sample size was limited, it is recommended that the median best represents the data (Altman, 1991). The chi‐square test was used to control for group differences between the Södertälje Young Adult Unit sample and Skåne County Council sample on sociodemographic characteristics. Group comparisons were made by the Mann–Whitney *U* test for ordinal and continuous variables and by chi‐square test for categorical variables, at baseline and outcomes at 12 months. This involved comparing the participants' characteristics and mental health assessment from the different sample groups at baseline, in which groups were viewed as potential predictors of the mental health outcomes. A logistic regression analysis was applied to calculate whether the younger (18–23 years) or older group (24–28 years) predicted mental health outcomes at 12 months. All potential predictors with an association at *p *= .10 or lower were entered in that analysis. The Wilcoxon signed rank test was used to assess changes of mental health over time, that is differences between sum scores at baseline, six and 12 months. Imputation principles were used for missing data, with the last observation carried forward (LOCF) or the next observation was carried backward (NOCB) (Fielding, Maclennan, Cook, & Ramsay, 2008). To describe the relationships and direction of the variables, we used correlation statistics with Spearman's ranking test. Values were measured; +1 or −1 indicated a perfect correlation and zero indicated no correlation. Effect size coefficients of variation (r) for correlations were calculated for non‐parametric data (Fritz, Morris, & Richler, 2012) and interpreted using Cohen's criteria (0.1 = small, 0.30 = medium, 0.50 = large) (Cohen, 1988). All statistical analyses were performed using SPSS version 23 (SPSS Inc., SPSS Armonk, NY, Released 2015, SPSS for Windows, Version 23.0). A two‐tailed p‐value of *p* ≤ .05 indicated statistical significance.

## 3 RESULTS

**Figure 1:**
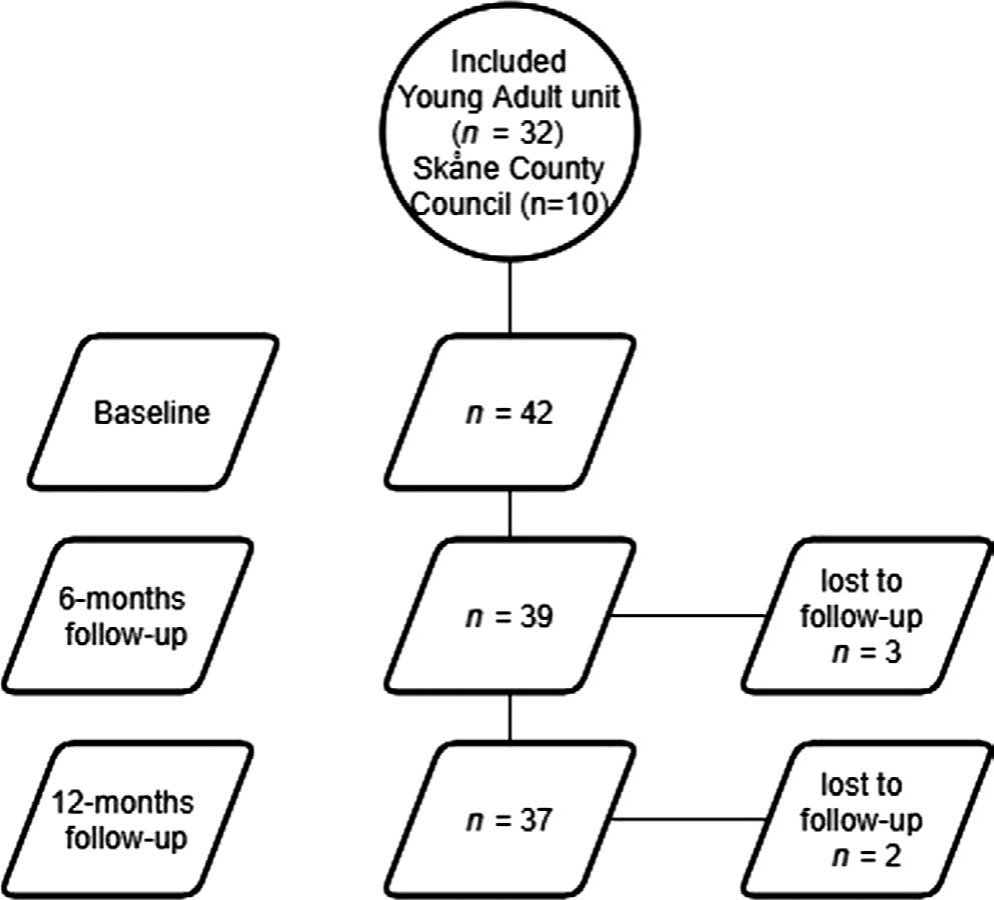
Study flow diagram

Forty‐two participants provided written consent and completed the baseline data collection. Three participants dropped out after baseline data collection and another two after the 6‐months follow‐up (Figure 1). These five participants chose not to continue due to personal reasons. Participant characteristics are presented in Table 1. Twenty‐seven were females, and fifteen were males. This distribution is consistent with National Board of Health and Welfare (2017) data on development of mental illness among children and young adults. Most participants were cohabiting with a partner or parents, had completed high school and had no income and originated from Sweden. The percentage of participants with major depressive disorders was 93% and 7% had bipolar disorder. The mean age at first contact with mental health services was 16 years. Twelve participants reported having no previous work experience. When performing group comparisons of the two age groups, statistically significant differences in sociodemographic characteristics were detected for age, age at first contact with mental health services, diagnosis and living situation. No differences were found in any of the mental health outcomes at baseline. At 12 months, a statistically significant difference between groups was detected about the outcome of engagement in everyday life activities, as assessed by POES (*p *= .004).

**Table 1:** Sociodemographic and clinical characteristics of the participants at baseline (*N* = 42)

Characteristics	Total *N* (%)	Young adult unit *N* (%)	Skåne County Council *N* (%)	*p*‐Value
Age				
Mean (min–max)	21 (18–28)	20 (18–23)	24 (20–28)	.000
Sex				
Women/men	27/15 (64/36)	21/11 (66/34)	6/4 (60/40)	.746
Diagnosis				
Depression/Bipolar disorder	39/3 (93/7)	32/‐ (100)/‐)	7/3 (70/30)	.003
Living situation				
Alone	7 (17)	3 (9)	4 (40)	.045
Cohabiting with partner or parents	29 (69)	25 (78)	4 (40)	
Other	6 (14)	4 (13)	2 (20)	
Education level				
Primary school	9 (21)	7 (22)	2 (20)	.918
High school/gymnasium level	26 (62)	20 (63)	6 (60)	
College/university	3 (7)	2 (6)	1 (10)	
Incomplete	4 (10)	3 (9)	1 (10)	
Age at first contact with mental health care (*N* = 41)				
Mean (min–max)	16 (8–26)	16 (9–21)	15 (8–26)	.253
Work experience (*N* = 35)				
Yes/no	23/12 (62/38)	15/10 (60/40)	8/2 (80/20)	.260
Income type				
Swedish Social Insurance Agency	15 (36)	8 (25)	7 (70)	.165
Swedish Board of Student Finance (CSN)	5 (12)	4 (13)	1 (10)	
Income support (municipality)	4 (10)	3 (9)	1 (10)	
None	17 (40)	16 (50)	1 (10)	

Note

The chi‐square and Mann‐Whitney test was used

The Wilcoxon signed rank test was used.

* *p* < .05

### 3.1 Changes in mental health outcomes

Changes in clinical recovery outcomes between measurement points are shown in Table 2. No statistically significant differences were found in depressive symptoms between baseline and follow‐up (*p *= .241), although a trend was noted. By the MADRS‐S definitions of depression severity, participants moved from having moderate to less severe depression between baseline and 12‐month follow‐up that showed a clinically improvement.

**Table 2:** Changes in outcome measurements between baseline, 6‐ and 12‐month follow‐up (*N* = 42)

Baseline (I)	6 months (II)			II–III	I–III
		I–II	12 months (III)		
Median (min–max)	Median (min–max)	*p*‐Value	Median (min–max)	*p*‐Value	*p*‐Value
Depression severity					
22 (3–42)	20 (0–35)	.194	20 (1–35)	.280	.24
Quality of life					
43 (20–68)	47 (24–75)	.051	49* (19–70)	.69	.007
Empowerment					
71 (52–93)	73 (56–97)	.109	74 (52–99)	.207	.075
Engagement in activities					
21 (13–34)	26* (12–36)	.013	26* (13–36)	.142	.002

Note

* *p* < .05.

Changes in personal recovery‐related outcomes about quality of life were calculated. A positive change in quality of life was noted between baseline and 12‐month follow‐up (*p *= .007). The median baseline score was 43, and this increased at 6‐month (MdN* = *47) and again at 12‐month follow‐up (MdN* = *49).

No statistically significant difference was found in empowerment between baseline and 12 months (*p *= .075). The baseline median score was 71, increased slightly at 6 months (MdN* = *73) and increased slightly again at 12 months (*MdN = *74), with scores neared significance. Before imputation of missing data, there was a statistically significant positive change between baseline and 12‐month data (*p *= .026).

Engagement in activities showed a statistically significant positive change from baseline to 12 months (*p *= .002). The baseline median score was 21 and had increased by five points at 12‐month follow‐up (MdN* = *26).

### 3.2 Association between outcomes

Correlations help describe the directions of relationship among the mental health variables at baseline and 12‐month follow‐up (Table 3). At baseline, depression severity and quality of life scores had a moderate correlation (*r_s_* = −0.384, *p *= .012) with 14% shared variance. At 12‐month follow‐up, the baseline association had increased to a 24% shared variance (*p *= .001).

**Table 3:** Correlation matrix for baseline and 12‐month follow‐up (*N* = 42)

	Depression severity		Quality of life		Empowerment		Occupational engagement	
	Baseline	12 months	Baseline	12 months	Baseline	12 months	Baseline	12 months
Depression severity			−0.384*	−0.495**	−0.544***	−0.361*	−0.292	−0.313*
Quality of life	−0.384*	−0.495**.			0.387*	0.427	0.443*	470*
Empowerment	−0.544***	−0.361*	0.387*	0.427			0.239	0.542
Occupational engagement	−0.292	−0.313*	.443*	0.470*	0.239	0.542		

Note

The Spearman's ranking test was used.

* *p* < .05

** *p* < .01

*** *p* < .001

Between depression severity and empowerment at baseline, a statistically significant correlation was present: greater perception of empowerment was correlated with a lower level of depression (*r_s_* = −0.544, *p *= .000). The association was weaker at 12‐month follow‐up (*r_s_* = −0.361, *p* = .019) and had a 13% shared variance. In the further analysis, no significant correlations could be found when examining the empowerment sum score in relation to other variables.

Higher level of engagement in activities at baseline had a moderate correlation with higher levels of quality of life (*r_s_* = 0.443, *p* = .003) with a 19% shared variance. At 12‐month follow‐up, the association had a 22% shared variance (*p* = .002). At 12‐month follow‐up, a higher level of engagement had a weak association with lower levels of depression severity (*p* = .044), with a 10% shared variance. At 6‐month follow‐up, all of the associations above were moderate and significant.

### 3.3 Binary logistic regression analysis

The variables engagement in everyday life activities (POES) and age were included in the regression model. Results showed that the odds of belonging to the group with higher engagement at 12 months increased in the older group of young adults. The odds ratio for engagement was 1.17 (95% CI 0.81–0.98), indicating that the odds increased, for an individual to belong to the older group. The Hosmer and Lemeshow goodness‐of‐fit test showed a *p*‐value of .244, indicating support for the model.

## 4 DISCUSSION

The results of the current longitudinal study indicate that the SSEE model of integrated mental health and vocational intervention for young adults improved quality of life and engagement in everyday activities among young adults with mental health problems. Improvement in empowerment scores from baseline and 12‐month follow‐up nearly met significance (*p* = .075). Depression scores decreased over the period studied and signalled clinically relevant changes. These findings are consistent with the findings from an earlier grounded theory study on young adults' process of work identity development by means of the so‐called Södertälje model (Liljeholm & Bejerholm, 2019) and helps develop a knowledge base of how to enhance the recovery process for young adults with mental health problems.

The earlier related study showed that the experience of receiving a person‐centred support that involved both mental health and vocational services towards employment contributed to a process of change where negative thought patterns transformed into more positive ones (Liljeholm & Bejerholm, 2019). The current study, which investigated clinical and personal recovery‐related outcomes of mental health, can be seen confirm these qualitative findings of more positive thought patterns with quantitative findings of a positive change in depression severity over 12 months. Both of these findings are in line with previous research on supportive employment for adults (Bejerholm et al., 2017; Corrigan, 2004). Therefore, being involved in work identity development may be important in mitigating depressive thoughts, increasing empowerment and thus better achieving clinical and personal recovery goals such as attaining work.

Furthermore, work identity development was connected to increased engagement, experience of more meaning and contributions to themselves and others, all of which affect how young adults develop self‐efficacy and long‐term future perspective (Liljeholm & Bejerholm, 2019). These attitudes are part of an essential process for understanding “me” that “I can” and starting to “believe in a future.” These positive changes in engagement in activities were confirmed by a statistically significant change of POES scores (Table 2). These results indicate that integrated mental health and vocational services that focus on enabling a working life for young adults decrease depressive thoughts, increase activity engagement, self‐efficacy and thus a future perspective. These are factors that are connected to both clinical and personal recovery (Anthony, 1993; Bejerholm & Roe, 2018; Bird et al., 2014). Further, the sample groups with different age ranges were shown to affect the average outcome of engagement at 12 months where the older participants had more engagement in activities. Probably, the difference may be due to that the older participants (40%) more often lived alone than the younger (7%) and had to engage in a wider range of everyday life activities connected to living an independent life.

Validation of the conceptual framework for personal recovery suggests that a new category, with greater emphasis on issues around diagnosis and medication, should be included in the original framework (Bird et al., 2014). In the current study, variables measuring clinical and personal recovery‐related outcomes were included. A trend was detected in the analysis of depression severity, with a change from moderate depression to less severe depression. The small sample size may be the reason a statistically significant change was not found. We think the assumption can be made that a clinically relevant change in decreased depression severity occurred. This decrease of depression may be of extra importance for young adults, since early interventions may reduce the risk of more severe problems in the future.

Empowerment was correlated with depression and quality of life at baseline. A moderate, inverse relationship was presented between empowerment and depression at 12‐month follow‐up. Previous return‐to‐work research shows that empowerment is inversely related to depressive symptoms (Bejerholm & Björkman, 2011; Porter & Bejerholm, 2018). This illustrates the complexity of measuring the recovery process when there seem to be two sides to the same phenomena. Depressive symptoms are a reflection of clinical recovery and empowerment outcomes reflect personal recovery. The correlation between better quality of life and fewer depressive symptoms at six and 12 months is explained as important components of a person's mental health. For example, housing, work and social life are also important quality of life components (Bejerholm et al., 2006; Priebe et al., 1999). When looking at the correlation matrix, the variables reflect a similarity between previous research on older adults who received integrated services and this supports the idea that this form of integrated service may be optimal for young adult recovery from mental problems.

The number of young adults with mental health problems is increasing and a group who are in great need of support in their recovery journey. This study confirms the potential of a vocational model with integrated mental health services for young adults' recovery processes, according to the SSEE model.

### 4.1 Implications for public health and mental health nurses

We believe that paying attention to both personal and clinical recovery in mental health services for young adults is important in providing an early chance at recovery. Efficient support in the transition towards a work identity provides a good condition for personal recovery from mental health problems among young adults, prevents exclusion from studies and work and lowers the risks for early exclusion in the community (Liljeholm & Bejerholm, 2019; Swedish Governments Official Investigations, 2018). These findings suggest that mental health services should include person‐centred and work‐oriented models, such as supported employment (Bond, Drake, & Becker, 2008) and supported education (Arbesman & Logsdon, 2011) for young adults. These efforts should not be seen as merely supporting employment, but rather as a natural part of a young adult's recovery process. A mental health service should not solely focus on so‐called clinical recovery with the main aim to foremost dampen psychiatric symptoms. Instead, psychiatric care should also offer support to help a client acquire essential life roles. Young adults do not need to fail with the normal change and transition to adulthood; instead, they need to be provided with the best conditions for early recovery from mental health problems at a young age. In line with the SSEE model, the recovery‐oriented services of Assertive Community Treatment model (ACT) and Flexible ACT (Marshall & Lockwood, 2011) are recommended that mental health services should be organized in teams of psychiatrists, nurses, psychologists, peer supporters and employment specialists and other specialists to pay attention to both care and support for social life roles concurrently and in relation to service users' personal goals (Bejerholm & Roe, 2018).

We believe that the integrated mental health and vocational services evident in the SSEE model can be applicable for mental health nurses and other professionals. The integrated SSEE model may support the professional development and clinical reasoning among nurses when working together with young adults who want to work and develop their work identity. In such a person‐centred practice, it is essential to attend young adults' resources, needs and preferences, when selecting interventions (Leamy et al., 2011). Of course, such conclusions and efforts towards recovery are already ongoing in the international context of public health and mental health services (Bejerholm & Roe, 2018; Bellack & Drapalski, 2012).

### 4.2 Methodological considerations

Alongside the emerging literature on recovery and the field of psychosocial research, being in recovery and achieving one's recovery goals is an individual journey that cannot be fully understood in relation to the concepts and measures used in the current study. This study was limited by a small sample size due to restricted opportunities for recruitment in middle‐sized city of Södertälje, but also Burlöv, Eslöv, Landskrona and Ängelholm in Sweden. The argument can therefore be made that the statistical trend of improved depression is clinically relevant. The small sample may also have affected the lack of correlation of empowerment with the other variables and the positive correlations. Analysing correlations between outcomes was not part of the initial study plan. However, these correlations contribute to a description of how different clinical and recovery‐oriented outcomes are related in young adults. The study can be said to be explorative and has provided with suggestive findings that can help when forming new assumptions for further research. The study design was chosen to quantitatively study the mental health changes indicated in a previous grounded theory study of the SSEE intervention. An experimental design with a control group would have strengthened the value of the result. No causal inferences could be drawn from the present study, although the temporality of the pre–post intervention design suggests that the SSEE intervention had an impact on outcomes. However, the risk for selection bias and contamination in the randomization process in the Södertälje context was too impending, who had a long record of providing integrated health and vocational services. Key strengths of the study are that the findings all point in the same direction and they confirm the quality of the study and value of integrated mental health and vocational support services for young adults.

## 5 CONCLUSION

The SSEE model of an integrated vocational and mental health intervention may result in changes in mental health that are linked to both personal and clinical recovery. The study suggests that the SSEE model has a significant statistical effect on quality of life and engagement in everyday activities. No significant statistical differences were found between measurement points with regard to depressive symptoms and empowerment; however, a positive trend was discerned. Altogether, the results suggest that it is of great importance to support young adults to improve their mental health and opportunities for employment, through both individual experiences of well‐being and in relation to a supporting social context (Bremberg & Dalman, 2015) and enhance recovery early on in their lives.

## CONFLICTS OF INTEREST

The authors report no conflicts of interest.

## ETHICAL APPROVAL

The first sample was derived from a randomized controlled trial of participants (*N* = 10, ages 20–26 years) in Scania County Council, which was approved by the Regional Ethical Review Board (Reg. no. 2011‐544) [33]. An additional ethical application was obtained for a sample in the city of Sodertalje, with a focus on young adults (*N* = 32, ages 18–26 years) (Reg. No. 2014‐277).
